# Sleep Quality of Hospitalized Patients, Contributing Factors, and Prevalence of Associated Disorders

**DOI:** 10.1155/2020/8518396

**Published:** 2020-01-20

**Authors:** Santi Kulpatcharapong, Pol Chewcharat, Kiat Ruxrungtham, Sutep Gonlachanvit, Tanisa Patcharatrakul, Busarakum Chaitusaney, Dittapol Muntham, Sirimon Reutrakul, Naricha Chirakalwasan

**Affiliations:** ^1^Department of Medicine, Faculty of Medicine, Chulalongkorn University, Bangkok, Thailand; ^2^King Chulalongkorn Memorial Hospital, Thai Red Cross Society, Bangkok, Thailand; ^3^Department of Anatomy, Faculty of Medicine, Chulalongkorn University, Bangkok, Thailand; ^4^Division of Allergy and Clinical Immunology, Department of Medicine, Faculty of Medicine, Chulalongkorn University, Bangkok, Thailand; ^5^Center of Excellence in Neurogastroenterology and Motility, Faculty of Medicine, Chulalongkorn University, Bangkok, Thailand; ^6^Excellence Center for Sleep Disorders, King Chulalongkorn Memorial Hospital, Thai Red Cross Society, Bangkok, Thailand; ^7^Section for Mathematics, Faculty of Science and Technology, Rajamangala University of Technology Suvarnabhumi, Phranakhon Si Ayutthaya, Thailand; ^8^Section of Endocrinology and Metabolism, Department of Medicine, Faculty of Medicine Ramathibodi Hospital, Mahidol University, Bangkok, Thailand; ^9^Division of Endocrinology, Diabetes and Metabolism, Department of Medicine, University of Illinois at Chicago, Chicago, Illinois, USA; ^10^Division of Pulmonary and Critical Care Medicine, Department of Medicine, Faculty of Medicine, Chulalongkorn University, Bangkok, Thailand

## Abstract

**Background:**

Data in the literature has shown poor sleep quality to be frequently observed in hospitalized patients and known to be associated with poor treatment outcome. Many factors may impact poor sleep quality, and there is currently limited available data. We aim to determine the prevalence of poor sleep quality and associated factors in patients admitted to internal medicine wards as well as the change of sleep quality over time after admission.

**Methods:**

An analytic observational study was conducted at the internal medicine wards at the King Chulalongkorn Memorial Hospital, Bangkok, Thailand. Patients were personally interviewed to evaluate the history of sleep quality at home, sleep quality after the first and the third days of admission, and potential associated factors. The Pittsburgh Sleep Quality Index and screening questionnaires for the common diseases associated with poor sleep quality were also utilized. The logistic regression analysis was used to determine the independent factors which led to poor sleep quality.

**Results:**

Data were collected from 96 patients during the period of June 2015 to February 2016. The mean age of the patients was 50.8 ± 16.7 years, and 51% were male. Infectious disease was the most common principal diagnosis accounted for 29.2%. The results show high prevalence of poor sleep quality after the first night of admission compared to baseline sleep quality at home (50% vs. 18.8%; *p* < 0.001). After 3 days of admission, the prevalence of poor sleep quality was reduced to the level close to baseline sleep quality at home (28.1% vs. 18.8%; *p* = 0.13). Multivariate analysis demonstrated that light exposure and pain were the main independent factors for poor sleep quality on the first day (odds ratio 6.68; 95% CI 2.25-19.84) and on the third day (odds ratio 3.47; 95% CI 1.24-9.71), respectively.

**Conclusions:**

This is the first study conducted on the sleep quality of hospitalized patients that included the follow-up period during hospital admission. Our study demonstrated high prevalence of poor sleep quality in hospitalized patients on the first day. Interestingly, the sleep quality was partly improved during hospitalization. Light exposure and pain were demonstrated to be the factors associated with poor sleep quality.

## 1. Introduction

Sleep is one of the basic activities of human daily living, and it affects human health physically and mentally [1]. In general, decreased sleep time during illness, stress, or change of a sleep environment such as hospitalization can affect sleep-wake cycle directly and causes daytime somnolence [2, 3].

From previous data, poor sleep quality was frequently observed in hospitalized patients resulting in patient concern [4, 5]. There are various factors that can lead to poor sleep quality including physical factors (e.g., disease, disease severity [4, 5], and pain [6, 7]), hospital environment (e.g., light and sound disturbance), doctor and nurse interruptions [8–11], and psychological factors (e.g., anxiety and stress) [12]. Poor sleep quality and sleep deprivation can affect many organ functions and health consequences including weakening immune system [13], increasing cardiovascular events [14], cognitive function impairment, and increased risk of falling and bone fracture in elderly [15].

Our primary objective was to determine prevalence of poor sleep quality in patients admitted to internal medicine wards and the adaptation of sleep qualities overtime after 3 days of admission. Our secondary objectives were to identify factors that contribute to poor sleep quality during admission including hospital factors (e.g., light exposure, sound exposure, and disturbance by nurses or doctors), patient factors (e.g., age, sex, underlying diseases, principle admission diagnosis, and the baseline sleep quality), and some associated diseases that can potentially lead to poor sleep quality including obstructive sleep apnea (OSA), allergic rhinitis (AR), and gastrointestinal disturbance.

## 2. Material and Methods

### 2.1. Study Design and Population

This analytic observational study was conducted at the internal medicine wards at King Chulalongkorn Memorial Hospital, Thai Red Cross Society, Bangkok, Thailand. Patients admitted to internal medicine ward who were at least 18 years of age were included in the study. The exclusion criteria were severe illness which required frequent vital sign monitoring more than every two hours, bedridden status, history of previous admission within 3 months, admission to an intensive care unit (ICU), condition that can disturb sleep-wake cycle including hepatic encephalopathy, uremic encephalopathy, toxic or substance intoxication/withdrawal, CNS infection, and patient refusal. All patients were personally interviewed using questionnaires to evaluate sleep quality at home, sleep quality at the first and the third nights of admission, and baseline clinical characteristics including age, sex, underlying disease, and current medication use. Questionnaires to screen for underlying disease that may affect sleep quality (OSA, AR, and gastrointestinal symptoms) were also utilized in the study. BMI was calculated from the measured height and weight upon admission.

### 2.2. Study Protocol

We used a stratified random sampling technique in sampling the patients from three different category wards of internal medicine including an ordinary ward (6-12 beds per room), a special ward (2-4 beds per room), and a private ward. After the first night of admission, patients were interviewed about their sleep quality at home, and sleep quality after the first day of admission, as well as their baseline clinical characteristics including age, sex, weight, height, alcohol and smoking habits, current medication, underlying diseases, and screening questions to identify the associated disease including OSA, AR, and gastrointestinal symptom as mentioned above. In addition, after the third day of admission, the sleep quality was reassessed using the same questionnaires. The patients were also asked to identify factors that disturb their sleep during admission including light exposure, sound exposure, disturbance by doctors, disturbance by nurses, stress, pain, and other factors (to be specified by the patient). Principal diagnosis was also obtained from a medical record.

Sleep quality at home, after the first night of admission, and at the third night of admission were all assessed by the questions modified from the Pittsburgh Sleep Quality Index (PSQI) which included six parameters: sleep latency (bedtime to time falling asleep), wake after sleep onset (total time awakening after falling asleep), early morning awakening (time from last awakening till wake up time), sleep efficiency (ratio of sleep duration to time spending in bed in percentage), use of sleeping pills, and patients' subjective sleep quality [16]. The questionnaire was validated in Thai language [17]. The poor sleep quality was determined by a subjective sleep quality score of at least 2 (0 = very good, 1 = fairly good, 2 = fairly bad, and 3 = very bad). We utilized the Berlin questionnaire to assess the risk of OSA [18]. This questionnaire was also validated in Thai language [19]. The Total Nasal Symptom Score (TNSS) was utilized to address the symptoms related to AR including nasal congestion, runny nose, nasal itching, and sneezing. Each symptom was categorized into 0 = no symptom, 1 = mild symptom, 2 = moderate symptom, and 3 = severe symptom. The total score of ≥3 was used to determine allergic rhinitis. Gastrointestinal symptoms were assessed using a set of questions on 16 symptoms (acid regurgitation, heartburn, regurgitation, chest tightness, globus symptom, dysphagia, epigastric burning, epigastrium fullness, nausea, vomiting, belching, anorexia, early satiety, bloating, constipation, and diarrhea) which may disturb the normal daily living and sleep quality. The severity of the symptom was categorized into mild, moderate, and severe. The presence of at least a moderate intensity of any symptoms was used to define gastrointestinal disturbance.

Sample size was calculated based on a previous study by Adobe-Hajbaghery et al. [20]. The study demonstrated that 220 out of 400 patients had poor sleep quality (55%) during hospitalization. The calculated sample size using a power of 80% and alpha of 0.05 led to the sample size of 96 patients.

### 2.3. Statistical Analysis

Baseline characteristics and all factors that affect the sleep quality were analyzed by a *t*-test or Mann-Whitney *U*-test for continuous data and a chi-square or Fisher's exact test for categorical data. In order to compare sleep quality before and after admission, a paired *t*-test was used for continuous data and McNemar's test for categorical data. The logistic regression analysis was used to determine the independent factor which led to poor sleep quality. The statistically significant difference was determined at *p* < 0.05. The statistical analysis was performed using SPSS version 22.

## 3. Results

During the period of June 2015 to February 2016, a total of 96 patients admitted to the internal medicine ward at King Chulalongkorn Memorial Hospital, Bangkok, Thailand, were enrolled in the study. 51%, 44.8%, and 4.2% were admitted to the ordinary ward, special ward, and private ward, respectively. The baseline characteristics and prevalence of poor sleep quality determined by a subjective sleep quality score of at least 2 are shown in Table 1. The mean age of our patients was 50.8 ± 16.7 years, and 51% of the patients were male. The three most common underlying diseases were hypertension (40.6%), cancer (23%), and type 2 diabetes mellitus (17.7%). The most common principal diagnosis was infectious disease which was accounted for 29% of patients. They included fever, septicemia, cellulitis, urinary tract infection, septic arthritis, spontaneous bacterial peritonitis, acute gastroenteritis, infective endocarditis, and dengue hemorrhagic fever. The second most common principal diagnoses were cardiopulmonary diseases and gastrointestinal diseases which accounted for 27.1% each. Cardiopulmonary diseases included congestive heart failure, pericardial effusion, acute asthmatic attack, pneumonia, acute bronchitis, COPD with acute exacerbation, and pneumothorax. Gastrointestinal diseases included upper and lower gastrointestinal bleeding, acute cholecystitis, acute cholangitis, common bile duct stone, liver abscess, hepatocellular carcinoma, pancreatic cancer, acute pancreatitis, and colorectal cancer. Hematologic diseases were accounted for 7.3% which included hemophilia A, snake bite, agranulocytosis, aplastic anemia, and lymphoma. Other diagnosis included anaphylaxis, nephrotic syndrome, hypoglycemia, lumbar spondylosis, adrenal insufficiency, Buerger's disease, and avascular necrosis of hip. The Berlin questionnaire identified 20.8% of our patients to have high risk for OSA. Allergic rhinitis was demonstrated in 14.6% using TNSS score ≥ 3. Gastrointestinal disturbance was identified in 12.5% of our patients using at least moderate intensity of any symptoms on the screening questionnaire.

As shown in Table 2, the prevalence of poor sleep quality using subjective sleep quality score ≥ 2 at the first night of admission was high at 50% compared to 18.8% at home (*p* < 0.001). Interestingly, after the third day of admission, the prevalence of poor sleep quality was remarkably reduced to 28.1% which was not statistically significantly different compared to home (*p* = 0.13). Moreover, if we defined the poor sleep quality using at least one of the criteria including sleep latency > 30 minutes, wake after sleep onset > 30 minutes, early morning awakening > 30 minutes, sleep efficiency < 85%, subjective sleep quality ≥ 2, and the use of sleeping pills; the prevalence of poor sleep quality after the first day of admission was demonstrated to be as high as 80.2% compared with 41.7% at home (*p* < 0.001), and it tended to improve to 59.4% after the third day of admission. Interestingly, when we separately analyzed the ordinary ward and special/private ward, only the ordinary ward demonstrated the statistically significant difference in terms of the prevalence of poor sleep quality on the third day of admission compared to home (*p* = 0.029 in an ordinary ward and *p* = 1.00 in a special/private ward) (Tables 3 and 4).

The prevalence of poor sleep quality among various principal diagnoses of the hospital admission including cardiopulmonary, infectious, gastrointestinal, hematologic, and other diseases were 57.7%, 35.7%, 55.7%, 71.4%, and 33%, respectively.

**Figure 1:**
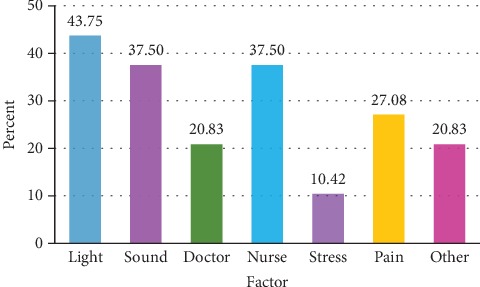
Factors reported in patients with poor sleep quality on the first day. ^∗^Other factors included cold temperature, hot temperature, cough, being in different place, itching, dyspnea, hungry, mosquito, polyuria, procedure, and disturbance by visitors.

Light exposure, sound exposure, and nurse disturbance were the most frequent factors reported by patients who had poor sleep quality on the first day (Figure 1). For predictors associated with poor sleep quality on the first day of admission, light exposure and the nurse disturbance were observed to be significant predictors for poor sleep quality using univariate analysis. After multivariate analysis, light exposure was the only parameter contributing to poor sleep quality in hospitalized patients (odds ratio 6.68; 95% CI 2.25-19.84) (Table 5).

**Figure 2:**
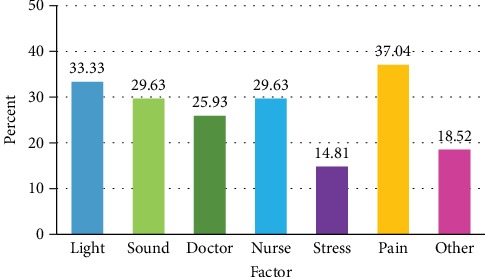
Factors reported in hospitalized patients with poor sleep quality on the third day. ^∗^Other factors included cold temperature, hot temperature, cough, being in different place, itching, dyspnea, hungry, mosquito, polyuria, procedure, and disturbance by visitors.

Pain, light exposure, sound exposure, and nurse disturbance were the most frequent factors reported by patients who had poor sleep quality on the third day (Figure 2). For predictors associated with poor sleep quality on the third day of admission, univariate and multivariate analyses revealed that pain was the sole parameter contributed to poor sleep quality for hospitalized patients (odds ratio 3.47; 95% CI 1.24-9.71) (Table 6).

None of the three diseases (OSA, AR, and gastrointestinal disturbance) were associated with poor sleep quality during admission.

## 4. Discussion

Our study demonstrated high prevalence of poor sleep quality (using subjective sleep quality) in approximately half of our hospitalized patients at the first night of admission. Our finding was similar to previously published studies which had shown the prevalence of poor sleep quality in hospitalized patients to be ranging between 43% and 91% [21–26] The sleep quality during admission worsened in all components including sleep latency, wake after sleep onset, early morning awakening, sleep efficiency, and subjective sleep quality. In addition, our study demonstrated that the sleep quality in hospitalized patients appeared to be improved after three days, particularly, sleep latency, sleep efficiency, and subjective sleep quality. We utilized subjective sleep quality as the primary parameter addressing sleep quality since the quality of sleep is primarily the patients' satisfaction with their sleep. A previous study also has demonstrated that subjective sleep quality not the objective sleep duration predicts next-day consequences [27]. An improvement of sleep quality after the third day of admission may be explained by familiarity of patients to the hospital environment and the improvement of medical illness. However, in the subgroup of patients admitted to the ordinary ward, the sleep quality remained poorer compared to home which supports the fact that a hospital environment likely affects the sleep quality in the hospital.

Many factors were known to contribute to poor sleep quality in in-patient setting such as patients' condition, environmental factors, or psychological factors [28, 29]. In our study, light disturbance was the factor that contributed to the poor sleep quality on the first day of admission, this finding corresponded to a previous study which demonstrated that light and noise exposures were the most disruptive factors contributing to poor sleep quality [30]. Light exposure can affect the patients' circadian rhythm which can cause sleep disturbance and lead to poor sleep quality. Light is known to suppress the release of melatonin, a sleep-promoting substance produced from the pineal gland controlled by the suprachiasmatic nuclei (SCN), the central circadian clock located in the hypothalamus [31]. Interestingly on the third day of admission, light exposure no longer appeared to be a significant predictor for poor sleep quality. This may be explained by personal adaptation and the impact of other more important factors. Pain was demonstrated to be the sole important factor of poor sleep quality on the third night. This finding is concurrent with the study demonstrating that pain was one of the most frequent reported reasons for poor sleep [25]. Furthermore, there was a previous study conducted in adolescents and their parents on the factors associated with sleep disturbances [32]. The study demonstrated that pain and somatic measures were associated with insomnia severity inventory (ISI) and PSQI scores after controlling for age, sex, depressive, and anxiety symptoms [32]. These findings highlight the importance of pain control in improving sleep quality.

In part of specific disease leading to hospital admission, the preliminary assumption was patients admitted for cardiopulmonary disease were likely to have poorer sleep quality because of the dyspnea symptom. However, we did not find the expected correlation. Our finding was similar to a previously published paper which demonstrated that admitting diagnosis was not associated with sleep duration or sleep quality in hospitalized patients [25]. Moreover, the comorbid diseases which could affect sleep quality including OSA, AR, and gastrointestinal disturbance also did not have association with poor sleep quality in our study.

Many studies have shown that sleep deprivation was associated with unfavorable medical condition, such as impaired host immune function, stimulating an inflammatory process of the body, impaired cognitive function, or even increased risk of coronary events [13, 14, 28]. Therefore, the poor sleep quality during hospitalization may have increased poor treatment outcome. Even though our study has shown that the sleep quality after a couple of days of admission was improved, the poor sleep quality at the first day of admission still could have affected the clinical complication. A further study may be needed to identify the relationship and the impact of the poor sleep quality during hospital admission in this specific group which only had poor sleep quality during the first day as demonstrated in our study. Since light exposure appeared to be the main culprit, we believe that the intervention to reduce light exposure is very important in order to improve sleep quality in hospitalized patients. Previous studies demonstrated the effectiveness of using eye mask in improving sleep quality in hospitalized patients in general medical wards, post anesthesia care units as well as in intensive care units [33–35]. A review also demonstrated that changing the sound and light environment appeared to be effective in improving sleep in hospitals [36]. Great variance of the sleep outcome reported, multitude of independent variables, and outcome metrics of the current available studies made it difficult to conclude. Further larger-scale studies in a more specific group of patients are still needed.

To our knowledge, our study is the first study conducted on sleep quality during admission with follow-up data during hospitalization and reported interesting findings as stated. However, we are aware that our study carries some limitations. Firstly, our study was conducted by an interview in which the patients who were unable to answer the questions were excluded. It is very likely that the patients with serious clinical illness were excluded from our study. Thus, the data demonstrated in our study may only represent the patients who had mild to moderate clinical illness. Secondly, our study was conducted using questionnaires to screen for OSA and to assess sleep quality in which standard polysomnography was not performed. Thirdly, our study was only conducted on the first day and the third day of hospital admission so the study with longer follow-up may give more insight on the true sleep quality affected by hospitalization.

## 5. Conclusion

This is the first study conducted on the sleep quality of hospitalized patients that included the follow-up period during hospital admission. Our study demonstrated a high prevalence of poor sleep quality in hospitalized patients, and interestingly, the sleep quality was partly improved during hospitalization. Light exposure and pain were demonstrated to be the factors associated with poor sleep quality.

## Figures and Tables

**Table 1:** Baseline clinical characteristics.

Character	*N* (%)
Sex	
Male	49 (51)
Female	47 (49)
Age (years, mean ± SD)	50.8 ± 16.7
Average BMI (kg/m^2^, mean ± SD)	22.9 ± 5.0
Underlying disease	
Hypothyroidism	1 (1)
Asthma	1 (1)
Anemia	4 (4.2)
Coronary artery disease	4 (4.2)
Stroke	4 (4.2)
Congestive heart failure	8 (8.3)
Chronic obstructive pulmonary disease	5 (5.2)
HIV	6 (6.25)
Chronic kidney disease	8 (8.3)
Type 2 diabetes mellitus	17 (17.7)
Cancer	22 (23)
Hypertension	39 (40.6)
Diagnosis	
Hematologic disease	7 (7.3)
Other	9 (9.4)
Cardiopulmonary disease	26 (27.05)
Gastrointestinal disease	26 (27.05)
Infectious disease	28 (29.2)
Ward	
Private ward	4 (4.2)
Special	43 (44.8)
Ordinary	49 (51)
Associated condition	
Gastrointestinal disturbance	12 (12.5)
Allergic rhinitis	14 (14.58)
Obstructive sleep apnea	20 (20.8)

**Table 2:** Prevalence of poor sleep quality (all wards).

	At home (%)	At the first night of admission		At the third night of admission		
		*N* (%)	*p* value	*N* (%)	*p* value (vs. at home)	*p* value (vs. day 1)
Sleep latency > 30 mins	16 (17)	38 (40)	<0.001^∗^	26 (27.3)	0.06	0.0192^∗^
Wake after sleep onset > 30 mins	11 (11.4)	32 (33.3)	<0.001^∗^	25 (26)	0.009^∗^	0.23
Early morning awakening > 30 mins	7 (7.4)	16 (16.8)	0.049^∗^	8 (8.3)	1	0.057
Sleep efficiency < 85%	23 (24.7)	54 (58)	<0.001^∗^	41 (43)	0.0039^∗^	0.016^∗^
Subjective sleep quality score ≥ 2	18 (18.8)	48 (50)	<0.001^∗^	27 (28.1)	0.13	<0.001^∗^
Use of sleeping pills	7 (7.3)	7 (7.3)	1	7 (7.3)	1	1
Total (at least one of poor sleep quality criteria)	40 (41.7)	77 (80.2)	<0.001^∗^	57 (59.4)	0.018^∗^	<0.001^∗^

∗*p* value < 0.05.

**Table 3:** Prevalence of poor sleep quality (ordinary ward).

	At home (%)	At the first night of admission		At the third night of admission		
		*N* (%)	*p* value	*N* (%)	*p* value (vs. at home)	*p* value (vs. day 1)
Sleep latency > 30 mins	6 (12.5)	19 (39.6)	0.007^∗^	13 (27.1)	0.071	0.157
Wake after sleep onset > 30 mins	4 (8.2)	21 (42.9)	<0.001^∗^	12 (24.5)	0.033^∗^	0.007^∗^
Early morning awakening > 30 mins	4 (8.3)	7 (14.3)	0.179	4 (8.3)	1.000	0.257
Sleep efficiency <85%	9 (19.1)	27 (57.4)	<0.001^∗^	21 (44.7)	0.005^∗^	0.109
Subjective sleep quality score ≥ 2	7 (14.3)	28 (57.1)	<0.001^∗^	16 (32.7)	0.029^∗^	0.003^∗^
Use of sleeping pills	4 (8.2)	3 (6.1)	0.563	3 (6.1)	0.563	1.000
Total (at least one of poor sleep quality criteria)	18 (36.7)	40 (81.6)	<0.001^∗^	29 (59.2)	0.022^∗^	0.008^∗^

∗*p* value < 0.05.

**Table 4:** Prevalence of poor sleep quality (special ward and private ward).

	At home (%)	At the first night of admission		At the third night of admission		
		*N* (%)	*p* value	*N* (%)	*p* value (vs. at home)	*p* value (vs. day 1)
Sleep latency > 30 mins	10 (21.3)	20 (42.6)	0.012^∗^	13 (27.7)	0.317	0.019^∗^
Wake after sleep onset > 30 mins	7 (14.9)	11 (23.4)	0.285	13 (27.7)	0.083	0.593
Early morning awakening > 30 mins	3 (6.4)	9 (19.1)	0.083	4 (8.5)	0.706	0.059
Sleep efficiency < 85%	14 (29.8)	28 (59.6)	0.003^∗^	20 (42.6)	0.157	0.046^∗^
Subjective sleep quality score ≥ 2	11 (23.4)	20 (42.6)	0.0495^∗^	11 (23.4)	1.000	0.020^∗^
Use of sleeping pills	3 (6.4)	4 (8.5)	0.563	4 (8.5)	0.654	1.000
Total (at least one of poor sleep quality criteria)	22 (46.8)	37 (78.7)	0.002^∗^	28 (59.6)	0.221	0.007^∗^

∗*p* value < 0.05.

**Table 5:** Predictors of poor sleep quality in hospitalized patients on the first day.

Predictor	Univariate analysis		Multivariate analysis	
	Odds ratio	*p* value	Odds ratio	*p* value
Light exposure	6.68 (2.25-19.84)	0.001	6.68 (2.25-19.84)	0.001
Nurse disturbance	2.6 (1.02-6.59)	0.044	—	—

**Table 6:** Predictors of poor sleep quality in hospitalized patients on the third day.

Predictor	Univariate/multivariate analysis	
	Odds ratio	*p* value
Pain	3.47 (1.24-9.71)	0.018

## Data Availability

Data are available upon request by contacting the corresponding author, Naricha Chirakalwasan, at narichac@hotmail.com.
